# Personalized Medicine in Gastrointestinal Diseases: From Molecular Insights to Precision Clinical Care

**DOI:** 10.3390/jpm16080399

**Published:** 2026-07-27

**Authors:** Athanasios Syllaios, Dimitrios Schizas

**Affiliations:** 1 Department of Colorectal Surgery, Addenbrooke’s Hospital, Cambridge University Hospitals, Cambridge CB2 0QQ, UK; 2 First Department of Surgery, National and Kapodistrian University of Athens, Laikon General Hospital, 11527 Athens, Greece; schizasad@gmail.com

## 1. Introduction

Gastrointestinal (GI) diseases comprise a heterogeneous group of disorders that collectively account for a substantial proportion of global morbidity, mortality, and healthcare expenditure. They encompass chronic inflammatory disorders, functional gastrointestinal conditions, metabolic diseases, and highly aggressive malignancies, each characterized by considerable biological and clinical heterogeneity. Despite significant advances in diagnostics and therapeutics over recent decades, conventional management strategies remain largely based on disease classification according to clinical presentation and histopathological findings. Such approaches frequently overlook the molecular complexity underlying disease pathogenesis, resulting in variable treatment responses, delayed diagnosis, unnecessary adverse effects, and suboptimal long-term outcomes.

The emergence of personalized medicine has fundamentally reshaped this paradigm by advocating for prevention, diagnosis, and treatment strategies tailored to the unique biological characteristics of individual patients. Rather than applying a “one-size-fits-all” approach, personalized medicine integrates genetic susceptibility, epigenetic regulation, transcriptomic and proteomic signatures, metabolomic profiles, environmental exposures, lifestyle factors, and the intestinal microbiome to guide clinical decision-making. The rapid evolution of next-generation sequencing, high-throughput multi-omics technologies, advanced imaging modalities, artificial intelligence (AI), and computational biology has accelerated the translation of these molecular insights into clinical practice, offering unprecedented opportunities to improve patient outcomes across the spectrum of gastrointestinal diseases [1].

## 2. Current Landscape of Personalized Medicine in Gastrointestinal Diseases

The implementation of precision medicine has been particularly transformative in gastrointestinal oncology, where comprehensive molecular profiling is increasingly integrated into routine clinical practice. Advances in next-generation sequencing have enabled the simultaneous identification of multiple actionable molecular alterations, facilitating individualized therapeutic strategies across colorectal, gastric, pancreatic, hepatobiliary, and oesophageal cancers. Beyond genomic profiling, growing evidence indicates that treatment response is also profoundly influenced by dynamic, non-genetic mechanisms of tumor adaptation. Chemoresistance is now recognized as an evolving process driven not only by clonal selection but also by epigenetically regulated cellular plasticity, in which drug-tolerant persister cells undergo transcriptional and chromatin remodeling, lineage reprogramming, and interactions with the tumor microenvironment that ultimately promote stable resistant phenotypes. These findings have highlighted epigenetic regulators as promising therapeutic targets and have stimulated the development of biomarker-driven strategies aimed at treatment priming, resensitization, and combination therapies [2]. Concurrently, increasing attention has focused on extracellular vesicles, particularly exosome-derived microRNAs (miRNAs), long non-coding RNAs (lncRNAs) and single nucleotide polymorphisms (SNPs), which actively mediate intercellular communication within the tumor microenvironment [3,4]. Rather than serving solely as biomarkers, these non-coding RNAs orchestrate complex signalling networks involved in epithelial–mesenchymal transition, cancer stemness, immune evasion, angiogenesis, metastatic dissemination, and therapeutic resistance, thereby contributing to tumor heterogeneity and disease progression. Their remarkable stability in biological fluids also supports their potential as minimally invasive biomarkers and therapeutic targets [3,4]. In parallel, liquid biopsy technologies, particularly circulating tumor DNA (ctDNA) analysis, have rapidly evolved into powerful tools for precision oncology, enabling early cancer detection, molecular residual disease assessment, longitudinal monitoring of treatment response, identification of emerging resistance mechanisms, and increasingly refined patient stratification. Together with advances in multi-omics integration, artificial intelligence, and real-world evidence, these innovations are reshaping the understanding of tumor biology and accelerating the transition toward more predictive, adaptive, and individualized management of gastrointestinal malignancies [5].

Beyond oncology, personalized medicine is rapidly transforming the management of chronic inflammatory gastrointestinal disorders, particularly inflammatory bowel disease (IBD), through a more comprehensive understanding of disease heterogeneity and the mechanisms underlying variable therapeutic responses. Advances in genomics, immune profiling, microbiome characterization, pharmacogenomics, and therapeutic drug monitoring are facilitating the transition from empirical treatment strategies toward biomarker-guided, individualized care. These approaches support more accurate patient stratification, optimization of treatment selection, prediction of therapeutic efficacy, and early identification of patients at increased risk of adverse drug reactions. Concurrently, the therapeutic landscape continues to expand with the development of next-generation biologics targeting key inflammatory pathways, including selective inhibition of interleukin (IL)-23 and other cytokine networks, as well as dual-targeting strategies designed to overcome treatment resistance in refractory disease. In parallel, microbiome-directed interventions, particularly fecal microbiota transplantation (FMT), are being actively investigated as novel approaches to restoring intestinal microbial homeostasis, especially in ulcerative colitis. Emerging regenerative and gene-based therapies, including mesenchymal stem cell therapy and CRISPR/Cas9-mediated genome editing, further exemplify the shift toward precision therapeutics by aiming to promote mucosal healing, restore immune homeostasis, and modify disease-driving molecular pathways [6].

One of the most rapidly expanding areas of personalized medicine is the characterization of the human gut microbiome. Mounting evidence demonstrates that alterations in microbial composition and function contribute to the development and progression of inflammatory, metabolic, and neoplastic gastrointestinal diseases. Integration of microbiome-derived biomarkers with host genomic and immunological data has opened new avenues for disease prediction, patient stratification, and individualized therapeutic interventions, including microbiota-targeted therapies, dietary modification, and precision nutrition. Although many of these approaches remain investigational, they illustrate the growing appreciation that host–microbe interactions represent a fundamental component of individualized gastrointestinal care [7].

Moreover, artificial intelligence and digital health technologies are revolutionizing multiple aspects of gastroenterology. Machine learning algorithms are increasingly incorporated into endoscopic imaging to improve adenoma detection, characterize mucosal lesions, and facilitate early cancer diagnosis. AI-assisted pathology, radiomics, and predictive analytics further complement molecular profiling by integrating diverse clinical datasets to generate individualized risk assessments and therapeutic recommendations. As these technologies mature, they are expected to enhance clinical efficiency while supporting more precise and data-driven decision-making [8].

## 3. Knowledge Gaps in Personalized Medicine for Gastrointestinal Diseases: Contributions of This Special Issue

Despite the remarkable progress achieved in personalized medicine, substantial scientific, technical, and clinical challenges continue to limit its widespread implementation across gastrointestinal diseases. Many proposed biomarkers remain insufficiently validated across diverse populations, while heterogeneity in study design, limited cohort sizes, and the lack of standardized methodologies for sample collection, sequencing, and data analysis continue to hinder reproducibility and clinical translation. Similar barriers are evident in microbiome research, where the considerable interindividual variability of the gut microbiota, together with differences in sequencing platforms and bioinformatic approaches, has limited the development of robust, clinically applicable microbiome-based biomarkers [9]. Nevertheless, accumulating evidence suggests that integrating host and microbial molecular data may substantially improve disease stratification and precision prevention. In this context, Lauricella et al. highlight the distinct host–microbiome landscape of early-onset colorectal cancer, demonstrating that this disease entity is characterized by unique microbial signatures and host–environment interactions that differ from those of late-onset colorectal cancer. Their work underscores the importance of multi-omics approaches integrating microbial composition with host genetic, immunological, and environmental factors to better understand disease heterogeneity and to facilitate the development of personalized diagnostic and therapeutic strategies [Contribution Lauricella et al.].

Another emerging area requiring further investigation is the role of mitochondrial biology in gastrointestinal diseases, particularly colorectal cancer. Increasing evidence suggests that mitochondrial metabolism extends beyond energy production to influence tumor progression, immune regulation, metabolic reprogramming, and therapeutic resistance. However, tumor metabolic plasticity and intratumoral heterogeneity continue to hamper the identification of reliable mitochondrial biomarkers and clinically effective therapeutic targets [10]. In this context, Desterke et al. demonstrated that mitochondrial and metabolic activities differ across molecular subtypes of colorectal cancer, with enhanced activity observed in MSI-high tumors and reduced activity in POLE-mutated and BRAF-V600E tumors. Their integrative transcriptomic and machine learning analyses further identified a metabolism–mitochondrial activity score associated with consensus molecular subtypes and predictive of BRAF-V600E mutation status, highlighting the potential of mitochondrial metabolism as a biomarker for tumor stratification, as well as a predictive biomarker for personalized therapeutic strategies [Contribution Desterke et al.].

Beyond gastrointestinal oncology, personalized medicine is also expanding our understanding of symptom heterogeneity in chronic liver diseases. Cholestatic pruritus remains one of the most debilitating complications of cholestatic liver diseases, substantially impairing patients’ quality of life despite recent therapeutic advances. Although current management follows a stepwise approach and novel therapies, including ileal bile acid transporter (IBAT) inhibitors, have expanded treatment options, the underlying pathophysiological mechanisms remain incompletely understood [11]. In this context, Beres et al. provide a timely overview of the evolving molecular landscape of cholestatic pruritus, highlighting the emerging role of the stratum corneum as a potential reservoir of circulating pruritogens. By integrating recent advances in non-invasive tape stripping with mass spectrometry-based lipidomic profiling, the authors discuss how cutaneous lipid signatures may facilitate biomarker discovery, improve understanding of disease mechanisms, and support the development of more targeted therapeutic strategies [Contribution Beres et al.]. Their work exemplifies the growing application of precision medicine approaches to hepatology, where molecular profiling has the potential to refine disease characterization and enable more individualized patient management.

The implementation of personalized medicine remains particularly challenging in the context of rare gastrointestinal diseases, where limited patient populations and the scarcity of robust prospective studies continue to impede the development of evidence-based, individualized therapeutic strategies. Although precision oncology has significantly improved outcomes in common gastrointestinal malignancies, patients with rare gastrointestinal tumors, uncommon disease entities, and infrequent postoperative complications remain underrepresented in clinical research, thereby limiting the generalisability of current precision frameworks. Addressing these gaps will require strengthened international collaboration, harmonised methodological approaches, multicentre registries, and the integration of real-world evidence to complement conventional clinical trial data. In this context, Papadakos et al. demonstrated that gastric cancers occurring in patients aged ≤24 years are typically diagnosed at advanced stages and are characterized by aggressive histopathological features, with prognosis primarily determined by tumor stage, histological subtype, and lymph node involvement, thereby providing clinically relevant risk stratification parameters for this understudied population [Contribution Papadakos et al.]. Similarly, Ziogou et al. highlighted the diagnostic and therapeutic complexity of primary gastric actinomycosis, an exceptionally rare gastrointestinal condition, emphasizing the need for individualized management strategies encompassing antimicrobial therapy, surgical intervention, or combined approaches [Contribution Ziogou et al.]. Furthermore, Tomara et al. analysed duodenal injuries following laparoscopic cholecystectomy, demonstrating that therapeutic decision-making—whether surgical, conservative, or endoscopic—should be tailored according to the timing of diagnosis, anatomical characteristics of the injury, and the patient’s clinical status [Contribution Tomara et al.]. Collectively, these studies underscore that, in rare gastrointestinal conditions and uncommon surgical complications, management strategies must remain highly individualized, reinforcing the broader necessity of precision-based, patient-specific approaches, even in low-prevalence disease settings.

## 4. Conclusions

The coming decade is expected to witness an accelerating convergence of molecular medicine, systems biology, artificial intelligence, digital health technologies, and real-world clinical data, collectively enabling increasingly precise and adaptive approaches to disease prevention, diagnosis, and treatment of gastrointestinal diseases. This integration will facilitate the transition from static, phenotype-based classifications toward dynamic, mechanism-driven disease definitions that incorporate longitudinal molecular, microbial, and clinical signatures. Continued collaboration among clinicians, molecular scientists, bioinformaticians, and healthcare policymakers will be essential to translate these advances into clinically meaningful and equitable improvements in patient care, while ensuring robustness, reproducibility, and scalability across healthcare systems.

Future progress in gastrointestinal personalized medicine will likely depend on the development of validated multi-omics biomarkers, standardized analytical frameworks, and interoperable data platforms capable of integrating genomic, transcriptomic, proteomic, metabolomic, microbiome, and imaging data into actionable clinical insights. The expansion of artificial intelligence and machine learning approaches is expected to further refine risk stratification, enable earlier disease detection, and support real-time therapeutic decision-making. At the same time, the incorporation of longitudinal real-world evidence will be critical for capturing treatment heterogeneity, evaluating long-term outcomes, and guiding adaptive treatment strategies in routine clinical practice.

Importantly, the success of personalized medicine in gastrointestinal diseases will also depend on addressing persistent barriers related to cost-effectiveness, data governance, ethical oversight, and equitable access to advanced diagnostics and targeted therapies. Without such considerations, there is a risk that the benefits of personalized medicine may remain concentrated in selected populations and high-resource settings. Ultimately, personalized medicine has the potential not only to optimize therapeutic efficacy but also to fundamentally redefine the practice of gastrointestinal medicine by shifting the paradigm from reactive disease management toward predictive, preventive, and continuously learning healthcare systems that are truly tailored to individual patients and evolving disease trajectories.

## Data Availability

No new data were created or analyzed in this study. Data sharing is not applicable to this article.
